# Effect of the patient information brochure in communicating the risks associated with crizotinib treatment to patients with non‐small cell lung cancer (NSCLC) in Europe

**DOI:** 10.1002/prp2.570

**Published:** 2020-03-31

**Authors:** Kui Huang, Terri Madison, Beatrice Wehler, Marcello Tiseo, Keith D. Wilner, Jingping Mo

**Affiliations:** ^1^ Epidemiology, Worldwide Safety and Regulatory Pfizer Inc New York NY USA; ^2^ Real World Strategy and Analytics Mapi, an ICON plc company Lexington KY USA; ^3^ Department of Radio‐Oncology University Hospital Saarland Homburg Germany; ^4^ 3rd Medical Department University Medical Center Mainz Germany; ^5^ Department of Medicine and Surgery University of Parma Parma Italy; ^6^ Medical Oncology Unit University Hospital of Parma Parma Italy; ^7^ Oncology Clinical Development Pfizer Inc La Jolla CA USA

**Keywords:** crizotinib, patient information brochure, risk minimization, survey

## Abstract

Crizotinib (XALKORI^®^) is indicated for anaplastic lymphoma kinase‐positive and ROS1‐positive advanced non‐small cell lung cancer. This study evaluated the distribution of the crizotinib patient information brochure (PIB) in Europe and patient knowledge of the key messages in the PIB. A cross‐sectional survey was conducted in 10 European countries among patients who received crizotinib to ascertain whether each patient received and read the PIB, and his/her knowledge of its key messages on hepatotoxicity, interstitial lung disease/pneumonitis, QTc prolongation, bradycardia, and vision disorders. Of the 341 patients contacted, 40 responded (11.7%), and 39 patients were eligible. A total of 77% of respondents acknowledged receiving the PIB, of which, 93% reported reading it. Knowledge of the individual side effects ranged from 36% to 85%, and precautions for use ranged from 56% to 67%. Understanding the reasons for calling a physician ranged from 54% to 85%. Knowledge of each of the 6 key side effects was greater among readers of the PIB compared to non‐readers or respondents who did not recall receiving the PIB. Approximately three‐quarters of survey respondents recalled receiving the crizotinib PIB and respondents who read the PIB were more knowledgeable of the key side effects of crizotinib than those who did not read or receive. Caution should be taken in generalizing these results because of the potential for selection bias and small sample size. These survey results suggest that the crizotinib PIB may be an effective risk communication tool for crizotinib‐treated patients in Europe.

**Abbreviations**
ALK: anaplastic lymphoma kinase
CI: confidence interval
EMA: European Medicines Agency
EU: European Union
ID: identification
ILD: interstitial lung disease
NSCLC: non‐small cell lung cancer
PIB: patient information brochure
PIL: patient information leaflet
RMM: risk minimization measure
UK: United Kingdom
US: United States

**Key Points**

- Approximately three‐quarters of survey respondents reported receiving the crizotinib patient information brochure (PIB)
- Respondents who received and read the PIB were more knowledgeable of the key side effects of crizotinib treatment than those who did not
- Results of this study suggest that the crizotinib PIB may be an effective risk communication tool for crizotinib‐treated patients in Europe

## 1 INTRODUCTION

Lung cancer is the leading cause of cancer‐related mortality worldwide.^1^ In 2018, the number of new lung cancer cases was estimated at 2.1 million worldwide, representing 11.6% of all new cancers, and the number of lung cancer deaths was 1.8 million, representing 18.4% of the total cancer deaths.^1^ In Europe, an estimated 470 039 new cases of lung cancer and 387 913 deaths occurred in 2018.^2^ Non‐small cell lung cancer (NSCLC) represents the majority of lung cancers (85%) and most commonly presents as inoperable locally advanced (Stage IIIB) or metastatic (Stage IV) disease. No curative treatment is currently available.^3^, ^4^Anaplastic lymphoma kinase (ALK)‐positive NSCLC constitutes a molecularly‐defined subgroup with an estimated prevalence of 2.7% of NSCLC.^5^

Crizotinib (XALKORI^®^) is an oral, small‐molecule tyrosine kinase inhibitor of ALK, MET and ROS1 receptor tyrosine kinases. In August 2011, crizotinib was approved in the United States (US) for the treatment of patients with ALK‐positive metastatic NSCLC. In October 2012, crizotinib was granted conditional approval in the European Union (EU) for the treatment of adults with previously treated ALK‐positive advanced NSCLC and was subsequently expanded to the first‐line treatment. Since 2016, the US and EU have approved crizotinib for the use in patients with ROS1‐positive NSCLC.

Crizotinib has been associated with a number of safety risks including hepatotoxicity, interstitial lung disease /pneumonitis, QTc prolongation, bradycardia, and vision disorders. The crizotinib label lists these risks as adverse reactions, and in Europe, these risks are included in the patient information leaflet (PIL). Additionally, Pfizer has developed educational materials in Europe as part of additional risk minimization measures (RMMs) requested by the Committee for Medicinal Products for Human Use (CHMP), which include a patient information brochure (PIB) to further inform patients receiving crizotinib treatment about known risks associated with crizotinib, as well as a patient identification (ID) card. The patient ID card, which includes spaces for patients to add their name, their oncologist's name, and the date crizotinib was started, is provided for patients to show their other healthcare providers.

This study was designed to assess the effectiveness of both the crizotinib PIB and patient ID card among EU patients. The specific objectives of the study were to assess patients' awareness, receipt, and use of the crizotinib PIB and patient ID card, and to assess if patients' knowledge of the key risks and actions required to minimize the key risks was in accordance with the information provided in these materials.

## 2 METHODS

### 2.1 Study design and population

This cross‐sectional study was conducted among crizotinib‐treated patients in Austria, Belgium, Denmark, France, Germany, Ireland, Italy, the Netherlands, Sweden, and the United Kingdom [UK]) from September 2014 to September 2016. Medical oncologists or pulmonologists in these 10 countries were contacted via a mailing list provided by the Intercontinental Marketing Services commercial database and was supplemented with information from local Pfizer Inc. country offices. The physicians were asked to recruit a convenience sample of patients receiving crizotinib.

Study eligibility criteria included treatment with crizotinib within 90 days prior to taking the survey, and completion of a signed and dated informed consent document, if applicable, based on each country's local regulations. Patients who participated in the survey pre‐testing and patients with immediate family members employed by Pfizer Inc, Mapi (the study vendor), or the European Medicines Agency (EMA) within the past 10 years were ineligible.

### 2.2 Survey instrument

The survey instrument included 35 yes/no or multiple‐choice questions plus four eligibility questions. Five questions focused on demographic characteristics. Seven questions assessed awareness, receipt, and use of the crizotinib PIB and patient ID card and four questions assessed how the PIB and ID card were received. Nineteen questions assessed the key patient‐directed risk messages for crizotinib including awareness of side effects, knowledge of precautions for use, and understanding of when to contact the physician; these were defined as effectiveness questions. Four of these effectiveness questions included four risks that were not related to crizotinib. The survey was designed to be completed in approximately 15 minutes.

The survey questionnaire underwent cognitive pre‐testing in each local language with 1 crizotinib‐treated patient each from Denmark, France, Germany, Italy, the Netherlands, and Sweden and 2 crizotinib‐treated patients from Belgium (1 for Belgium in French, the other in Flemish). This approach was based on feasibility considerations. From a feasibility perspective, there were constraints due to the inability to directly identify patients who received crizotinib given ethical considerations and privacy regulations. Pre‐testing was not conducted in Austria since the instrument had been tested in German. Since the European Medicines Agency (EMA) had endorsed the English version of the questionnaire, it was not pre‐tested in Ireland or the UK. Pfizer offices in Austria, Ireland, and the UK reviewed the survey questionnaire to confirm that terminology used was consistent with local crizotinib educational materials.

Experienced personnel in cognitive pre‐testing and linguistic validation of survey questionnaires conducted pre‐testing with 1‐on‐1 interviews. The cognitive pre‐test informed necessary minor revisions to most of the country‐specific versions of the patient questionnaire, mainly modifications of the initial translations to conform to local standards or language nuances (eg, in some countries, “true/false” was more commonly communicated as “yes/no”). Other changes identified from pre‐testing included:

- The generic name for XALKORI® (ie, crizotinib) was provided throughout the survey because the brand name was not recognized by several patients.
- The French translation of instructions regarding side effects associated with XALKORI® was revised to make clear that the purpose was to inquire about information the respondent learned from the PIB rather than to collect information about side effects personally experienced

Surveys were self‐administered in local languages either via the internet or paper, depending on respondent's preference. Confirmit, a software platform designed specifically for surveys, was used to administer the survey and collect data.

### 2.3 Survey administration

After securing any required local approvals (such as ethics committees), physicians who were willing to recruit patients for the survey were provided with patient survey kits. The contents of the patient survey kit were:

- a letter to patients inviting participation in the survey which included study details, a unique code, and instructions for online access of the survey,
- for all countries except France, an informed consent document; in France, a study information document conforming to local regulations in lieu of the informed consent,
- a paper survey with the same unique code to be completed by the patient and a postage paid, pre‐addressed envelope for returning the paper survey, if the paper survey method was chosen by the patient.

The number of completed surveys was tracked to monitor progress to identify study sites and physicians with no or few surveys completed by patients.

### 2.4 Data analysis

The dataset for analysis comprised all eligible patients who answered at least 1 of the survey questions about the effectiveness of the additional RMMs for crizotinib. SAS^®^ version 9.2 (SAS Institute, Cary, NC) was used for all analyses. The absolute and relative frequency (%) of each category and number of missing data were described with qualitative variables. Two‐sided 95% confidence intervals (CI) for percentages were determined for the effectiveness endpoints using exact methods.

## 3 RESULTS

Among the 10 countries that participated, 56 study sites or physicians received 341 survey kits to provide to the patients. A total of 40 patients were recruited by physicians, yielding a survey response rate of 11.7% (40/341). Thirty‐nine of the 40 patients who met the study eligibility requirements and answered at least 1 main question of the survey were included in the analysis. No surveys were received from patients in Denmark, Ireland, or the UK.

The characteristics of NSCLC patients who responded to the survey are presented in Table 1. Most respondents were female (72%), were <65 years of age (77%), and had crizotinib treatment within the past month (82%). Most patients (57%) were not currently participants in a clinical trial of crizotinib. A total of 33% of respondents had completed university/higher education.

**Table 1:** Characteristics of survey respondents

	Overall (N = 39)
	n (%)
Country of Origin	
Austria	2 (5)
Belgium	5 (13)
Denmark	0 (0)
France	1 (3)
Germany	9 (23)
Ireland	0 (0)
Italy	17 (43)
Netherlands	1 (3)
Sweden	4 (10)
United Kingdom	0 (0)
Last time treated with crizotinib	
Within the last month	32 (82)
1 month ago	2 (5)
2 months ago	3 (8)
3 months ago	2 (5)
I don't know	0 (0)
Current participant in a crizotinib clinical trial	
Yes	11 (28)
No	22 (57)
I don't know	6 (15)
Gender	
Male	11 (28)
Female	28 (72)
Age group	
18‐44	9 (23)
45‐54	8 (21)
55‐64	13 (33)
65‐74	7 (18)
75 or older	2 (5)
Educational level	
Primary school	7 (18)
Secondary school	11 (28)
University/higher education	13 (33)
Prefer not to answer	1 (3)
Missing Data	7 (18)

Although only 49% (n = 19) of respondents stated awareness of the PIB for crizotinib, 77% (n = 30) acknowledged PIB receipt. A total of 93% (n = 28) of respondents who recall receiving the PIB said they read it. Among the 14 respondents who indicated awareness of the patient ID card, 21% reported using it.

Familiarity with the key crizotinib side effects ranged from 36% to 85% (Table 2). Most respondents expressed knowledge of “changes to vision” (85%), “dizziness, light‐headedness, fainting, tiredness” (69%), and “abnormalities in liver blood tests” (61%). Nearly half (49%) of respondents knew crizotinib was associated with “chest discomfort or irregular heartbeat.” More than a third of respondents knew that crizotinib may “slow heart rate” (38%) or could cause “breathing problems” (36%).

**Table 2:** Proportion of survey respondents knowledgeable of each key message^a^

Key message	Overall (N = 39)	
	n (%)	95% CI
Side effects		
Breathing problems (Q1A)	14 (36)	[21; 53]
Abnormalities in liver blood tests (Q1B)	24 (61)	[45; 77]
Dizziness, light‐headedness, fainting, tiredness (Q1D)	27 (69)	[52; 83]
Chest discomfort or irregular heartbeat (Q1F)	19 (49)	[32; 65]
Changes to vision (Q1G)	33 (85)	[69; 94]
Slow in heart rate (Q1H)	15 (38)	[23; 55]
Precautions for use		
May need to stop driving or operating machinery for vision changes (Q2A)	26 (67)	[50; 81]
Inform your doctor of persistent or worsening changes to vision (Q2B)	27 (69)	[52; 83]
Doctor will monitor your heart function and may adjust your crizotinib dosage (Q2C)	22 (56)	[40; 72]
Reasons to call your doctor		
Light‐headedness, chest discomfort, fainting (Q3A)	33 (85)	[69; 94]
Skin and whites of your eyes turn yellow (Q3B)	26 (67)	[50; 81]
Urine turns dark or brown (tea colour) (Q3C)	22 (56)	[40; 72]
Nausea, vomiting (Q3D)	29 (74)	[58; 87]
Difficulties with breathing, cough, fever (Q3E)	33 (85)	[69; 94]
Itching, or bruised more easily than usual (Q3F)	21 (54)	[37; 70]

a Results of four risks not related to crizotinib are not included in the table.

In general, knowledge of precautions for crizotinib use was higher than knowledge of side effects. About two‐thirds of respondents reported knowledge of the necessity of stopping driving and operating machinery for changes in vision (67%) or informing their physician about persistent or worsening visual changes (69%). More than half of respondents (56%) reported knowledge that their heart function would be monitored by their doctor and that the dosage of crizotinib might require adjustment.

The range of understanding of reasons to contact the physician ranged from 54% to 85%. Specifically, the rates of knowledge of when to contact the physician for “light‐headedness, chest discomfort, fainting” and “difficulties with breathing, cough, fever” were 85%. Most respondents (74%) reported knowing to contact the physician for “nausea, vomiting,” 67% knew to contact the physician for “skin and whites of your eyes turn yellow,” 56% knew to contact the physician for “urine turns dark or brown (tea colour)”, and 54% knew to contact the physician for “itching, or bruised more easily than usual.”

**Figure 1:**
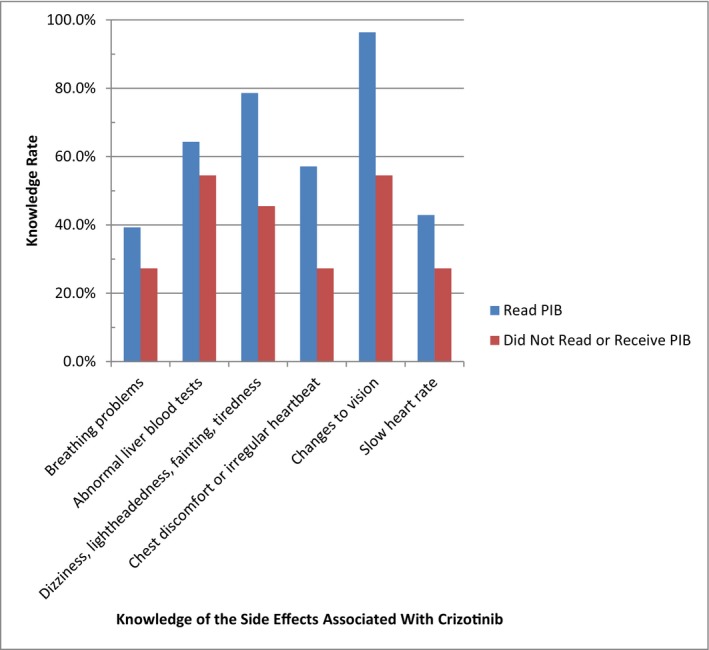
Effect of reading the PIB on survey respondents' knowledge of each key side effect associated with crizotinib treatment

Knowledge of individual side effects stratified by respondents who did and did not read or receive the PIB is presented in Figure 1. Respondents who reported reading the PIB were more knowledgeable of each of the 6 key side effects than respondents who did not read or receive it.

## 4 DISCUSSION

In our study, most patient respondents reported receiving the PIB (77%), of which 93% reported reading it. Our results are similar to results reported in the limited number of studies identified in the literature that directly evaluated the effectiveness of RMMs with patients.^6^, ^7^, ^8^ Specifically, levels of receipt/reading of patient‐directed RMMs in other studies were as follows: 88%/93% (Landsberg et al^6^), 93%/86% (Enger et al^7^), and 89%/86% (Brandenburg et al^8^). Awareness of the side effects and precautions for use of crizotinib, and reasons for contacting the physician was high for vision‐, hepatic‐, and dizziness‐related items, with knowledge levels ranging from 61% to 85%. However, fewer than half of respondents knew crizotinib was associated with “chest discomfort or irregular heartbeat,” “slow in heart rate,” or “breathing problems.” Knowledge levels in our study were similar or higher to those reported by Landsberg et al, where knowledge levels of risks associated with aripiprazole ranged from 46% to 69% and knowledge levels of behaviors in case these risks occur was 56%‐69%.^6^ Similarly, a study that evaluated knowledge levels of precautions for use for, and risks associated with, varenicline reported patient knowledge levels of 19%‐82%.^7^ Knowledge levels in our study were lower than those reported by Brandenburg et al, where knowledge levels of teratogenic‐related risks for lenalidomide and thalidomide assessed by 5 survey questions ranged from 87% to 98%.^8^ However, it is not surprising that knowledge levels for teratogenic‐related risks were high, given the severity of this risk as well as the restricted distribution REMS program in the US specifically employed to minimize teratogenic risks associated with lenalidomide and thalidomide.

Ideally, the effectiveness of RMM should be measured against a comparator group not exposed to the RMM. However, such a comparator group is not possible for medicines that have RMM required at the time of initial authorization.^9^ In order to circumvent this limitation, we considered levels of knowledge for all 6 key side effects by whether patients reported having read or received the PIB or not. In this study, respondents who read the PIB showed consistently greater knowledge for all 6 key side effects than respondents who did not read or receive, suggesting the PIB was a useful supplement to the PIL in communicating the risks associated with crizotinib to patients. These results are consistent with those reported by Enger et al, where patients who read the varenicline medication guide had higher knowledge levels in comparison with patients who did not.^7^

A key limitation to our study was the low number of completed surveys, where only 40 patients responded to the survey. The target NSCLC patient population is generally one that has a terminal diagnosis and poor prognosis. Despite improvements in overall survival for patients with ALK‐positive NSCLC who are treated with crizotinib, patients diagnosed with NSCLC, in general, have an average life expectancy of ranging from 10 to 26.7 months,^10^ and this may have contributed to the relatively low response rate. The response rate was not as low as the other EU‐based survey we found in the literature, where only 16 patients/caregivers participated in the survey conducted by Landsberg et al.^6^ Enger et al reported a response rate of 18%, however, this US‐based study allowed identification of specific patients receiving varenicline from a large healthcare claims database whereas in Europe, a similar approach is not allowed due to stringent European privacy regulations. Similarly, due to the restricted distribution Risk Evaluation and Mitigation Program in place for lenalidomide and thalidomide, Brandenburg et al were able to fully identify all US‐based patients receiving these medicines, yet still reported a relatively low response rate of 3.8%.

Since ALK‐positive NSCLC is relatively rare, the number of physicians prescribing crizotinib is expected to be low. Our study faced recruitment challenges largely because of the paucity of treating physicians who were available to identify patients for survey participation, and feasibility limitations such as the undue lengthy processes for ethics approvals in some countries without prior experience with surveys that evaluate the effectiveness of RMMs. Hence, based on the confidence interval for one proportion with exact (Clopper‐Pearson) formula, the small number of patients who completed the survey (n = 39) resulted in low precision of the knowledge rate estimates (11.9%–15.5%). Similar to recommendations provided by Landsberg et al,^6^ we suggest that additional guidance from regulatory agencies is needed to improve the collaboration between the industry and patient stakeholders to facilitate wider reach to assess the clinical importance of patient‐directed interventions such as RMMs.

Our study used a convenience sample, which could have introduced a selection bias. We sought to limit this effect by including countries with the highest crizotinib prescribing rates and obtaining a diverse sample of multiple regions of the EU. Of note, in the survey conducted by Landsberg et al, a randomized approach was still not successful in recruiting a representative sample, and only 16 patients/caregivers responded from only 3 of 12 participating countries in their survey.^6^ However, caution is advised in generalizing the results to all patients in the EU as the number of study respondents was small across the 7 countries.

Information bias may have also affected knowledge rates in this study. To minimize this bias, the online version of the survey was designed with randomized response sets for all multiple‐choice questions. Additionally, patients were also asked to complete the survey in a single sitting to minimize the possibility of searching for the correct answers. Answers to questions were not able to be revised on the on‐line version of the survey. If the survey was completed at the physician's office, the physician was instructed not to request that patients to clarify or revise their survey responses. Despite these efforts in study design, differential misclassification bias was possibly an issue for the 7 questions regarding “for which of the following should you call your doctor right away while taking XALKORI®.” Most respondents answered “yes” for all 7 questions, regardless of whether or not PIB included the listed risks. Any of the listed risks, regardless of an association with crizotinib, would likely prompt patients to contact their physician. In retrospect, had the question had been worded as “for which of the following does the crizotinib PIB recommend for you to call your doctor right away while taking crizotinib,” the 7 questions may have provided more useful knowledge about the information specific to the PIB. An additional source of potential bias is the use of self‐reporting. Although self‐reporting is the only way to assess a person's knowledge, patient‐reported information may still be subject to recall bias.

Overall, the survey results suggest that most patients who responded to the survey received and read the crizotinib PIB. The majority of patients who responded to the survey were aware of crizotinib side effects, precautions for use, and reasons to contact the physician. Knowledge rates were consistently greater among patients who read the PIB compared with patients who did not read or receive the PIB. The findings of this survey suggest that the PIB may be an effective way to provide information about risks to patients receiving crizotinib.

## CONFLICT OF INTEREST

Terri Madison is an employee of Mapi, an ICON plc Company, who received funding from Pfizer Inc. to conduct this study and to develop this manuscript. Kui Huang, Jingping Mo, and Keith Wilner are employees of Pfizer Inc. which commercializes crizotinib in Europe.

## AUTHOR CONTRIBUTIONS

Kui Huang, Terri Madison, and Jingping Mo contributed to the study design; Beatrice Wehler and Marcello Tiseo contributed to data collection; Kui Huang, Terri Madison, Keith Wilner, and Jingping Mo contributed to data analyses; Terri Madison and Kui Huang drafted the manuscript; Beatrice Wehler, Marcello Tiseo, Keith Wilner, and Jingping Mo reviewed the manuscript.

## References

[1] Bray F , Ferlay J , Soerjomataram I , Siegel RL , Torre LA , Jemal A . Global cancer statistics 2018: GLOBOCAN estimates of incidence and mortality worldwide for 36 cancers in 185 countries. CA Cancer J Clin. 2018;68(6):394‐424. Available at: https://onlinelibrary.wiley.com/doi/full/10.3322/caac.21492. Accessed February 20, 2020. 30207593 10.3322/caac.21492

[2] IARC 2018. http://gco.iarc.fr/today/data/factsheets/cancers/15-Lung-fact-sheet.pdf (accessed 01 November 2018).

[3] Cancer Research UK. http://www.cancerresearchuk.org/about-cancer/lung-cancer/stages-types-grades/types (accessed 01 December 2017).

[4] Howlader N , Noone AM , Krapcho M , et al. (eds). SEER Cancer Statistics Review, 1975–2013, National Cancer Institute. Bethesda, MD, http://seer.cancer.gov/csr/1975_2013/, based on November 2015 SEER data submission, posted to the SEER web site, April 2016.

[5] Varella‐Garcia M , Cho Y , Lu X , et al. ALK gene rearrangements in unselected caucasians with non‐small cell lung carcinoma (NSCLC). J Clin Oncol. 2010;28(15_suppl):10533.

[6] Landsberg W , Al‐Dakkak I , Coppin‐Renz A , et al. Effectiveness evaluation of additional risk minimization measures for adolescent use of aripiprazole in the European Union: results from a post‐authorization safety study. Drug Saf. 2018;41:797‐806. 29671224 10.1007/s40264-018-0662-2 PMC6061424

[7] Enger C , Younus M , Petronis KR , Mo J , Gately R , Seeger JD . The effectiveness of varenicline medication guide for conveying safety information to patients: a REMS assessment survey. Pharmacoepidemiol Drug Saf. 2013;22:705‐715. 23349095 10.1002/pds.3400

[8] Brandenburg NA , Bwire R , Freeman J , Houn F , Sheehan P , Zeldis JB . Effectiveness of risk evaluation and mitigation strategies (REMS) for lenalidomide and thalidomide: patient comprehension and knowledge retention. Drug Saf. 2017;40:333‐341. 28074423 10.1007/s40264-016-0501-2 PMC5362654

[9] Banerjee AK , Zomerdijk IM , Wooder S , Ingate S , Mayall SJ . Post‐approval evaluation of effectiveness of risk minimisation: methods, challenges and interpretation. Drug Saf. 2014;37(1):33‐42. 24357107 10.1007/s40264-013-0126-7

[10] Lee DH , Tsao M‐S , Kambartel K‐O , et al. Molecular testing and treatment patterns for patients with advanced non‐small cell lung cancer: PIvOTAL observational study. Ahmad A, ed. PLoS ONE. 2018;13(8):e0202865 10.1371/journal.pone.0202865. 30148862 PMC6110501

